# Physiological Properties and *Salmonella* Growth Inhibition of Probiotic *Bacillus* Strains Isolated from Environmental and Poultry Sources

**DOI:** 10.1155/2013/958408

**Published:** 2013-05-26

**Authors:** Anita Menconi, Marion J. Morgan, Neil R. Pumford, Billy M. Hargis, Guillermo Tellez

**Affiliations:** Department of Poultry Science, University of Arkansas, Fayetteville, AR 72701, USA

## Abstract

The objective of the present study was to describe the physiological properties of seven potential probiotic strains of *Bacillus* spp. Isolates were characterized morphologically, biochemically, and by 16S rRNA sequence analyses for identification. Tolerance to acidic pH, high osmotic concentrations of NaCl, and bile salts were tested. Isolates were also evaluated for their ability to metabolize different carbohydrates sources. The antimicrobial sensitivity profiles were determined. Inhibition of gastrointestinal *Salmonella* colonization in an avian model was also evaluated. Five strains of *Bacillus* were tolerant to acidic conditions (pH 2.0) and all strains were tolerant to a high osmotic pressure (NaCl at 6.5%). Moreover, all strains were able to tolerate concentration of 0.037% bile salts after 24 h of incubation. Three strains were able to significantly reduce *Salmonella* Typhimurium levels in the crop and in the ceca of broiler-type chickens. Among the 12 antibiotics tested for antibiotic resistance, all strains were resistant to bacitracin and susceptible to gentamycin, neomycin, ormethoprim, triple sulfa, and spectinomycin. Bacterial spore formers have been shown to prevent gastrointestinal diseases in animals and humans. The results obtained in this study show important characteristics to be evaluated when selecting *Bacillus* spp. candidates to be used as probiotics.

## 1. Introduction

Probiotics have been commercialized for both animal and human uses. Probiotics for humans use are subject to minimal restrictions and come in many different forms. Probiotics in animal feed have been used for the prevention of gastrointestinal infections, with a wide use in poultry and aquaculture productions [1–6]. 

Diarrhea is one of the major side effects of chemotherapy in cancer treatments and has been associated with increased morbidity, mortality, increased treatment costs, and restrictions related to the ability to deliver full doses of chemotherapy [7, 8]. Enterocyte proliferation in the intestinal mucosa and the intestinal microflora can be directly harmed by the effect of chemotherapeutic agents as well as radiation, often causing bacterial translocation, malabsorption, and/or diarrhea [8, 9]. Therefore, in order to reduce systemic bacterial diseases, high doses of broad spectrum antibiotics are usually used in cancer patients undergoing chemotherapy or radiation therapy. The disruption of the beneficial intestinal microflora is a common consequence to this type of treatment, which may lead to the colonization of opportunistic pathogenic bacteria such as *Salmonella* spp. [10, 11] and *Clostridium difficile* [12, 13]. Although the most common types of probiotics available are based on lactic acid bacteria (LAB), there are other potentially beneficial microorganisms that are not normally found in the gastrointestinal tract (GIT) such as *Saccharomyces boulardii *or *Bacillus *spp. For example, *Saccharomyces boulardii* has been shown to prevent the recurrence of *Clostridium difficile*-induced pseudomembranous colitis [14] as well as *Escherichia coli* infections [15]. Spore-forming bacteria such as *Bacillus subtilis*, *B. megaterium*, *B. licheniformis*, *Paenibacillus polymyxa*, and *B. clausii* have also been used as probiotics in humans [1]. 

Many studies have shown that either strains of live bacteria or active spores can efficaciously reach the intestine, preventing colon carcinogenesis [16, 17]. Moreover, they can suppress the development of preneoplastic lesions [18]. These microorganisms can also release antimicrobial substances active against Gram-positive bacteria such as *Staphylococcus aureus*, *Enterococcus faecium*, and *Clostridium difficile* and can induce IFN-gamma production and CD4+ T-cell proliferation [19, 20]. Products containing *Bacillus* spp. spores are used commercially as probiotics because they have some advantages over the traditional LAB products, for example, the ability to be stored indefinitely in a dry form [1, 4, 21, 22] and the ability to survive baking processes [23]. 

Current research has shown that *Bacillus subtilis* spores, after oral ingestion, are immunogenic and are able to disseminate to Peyer's patches and mesenteric lymph nodes [23–25]. Three main findings have supported the hypothesis that *Bacillus subtilis* spores can germinate in the small intestine. First, following oral ingestion in mice, Hoa et al. [26] showed that more *Bacillus subtilis* spores were excreted after ingestion than initially given. Second, after administration of spores to mice, expressed mRNA of vegetative cells was detected in the GIT by reverse transcription (RT)-PCR [27]. Third, after oral administration of spores to mice, systemic immunoglobulin G was produced against vegetative *Bacillus subtilis* cells [24]. The above studies indicate that *Bacillus* spp. spores are not merely present in the intestinal tract as transient bacteria, but they might also have some interaction with the host enterocytes, immunocompetent cells, or with the intestinal microbiota [22].

Identifying desirable physiological properties and the ability to inhibit the growth of pathogenic bacteria are very important when selecting potential candidates to be used as probiotics for humans and animals. In the present study, *Bacillus* spp. strains, isolated from poultry and environmental sources, were characterized and evaluated for their ability to metabolize different carbohydrate sources, their antibiotic sensitivity profile, and their tolerance to acidic pH, high osmotic concentrations of sodium chloride (NaCl), and bile salts. In addition, inhibition of *Salmonella* colonization in a well-established avian model was also evaluated.

## 2. Materials and Methods

### 2.1. Isolation, Biochemical Tests, and Identification of Selected *Bacillus* Strains

Strains* of Bacillus *spp., laboratory identified as NP122, AM0904, B2, RW41, AM0902, AM1109A, and AM1109B, were isolated from environmental and poultry sources as described by Wolfenden et al. [28]. Biochemical evaluation tests as well as identification for these seven selected strains were carried out using a bioMerieux API 50 CHB test kit (catalog number 50430, bioMerieux, Marcy l'Etoile, France). The identification procedure, which followed the manufacturer's instructions, was also important to confirm generally recognized as safe (GRAS) status of the isolates. Besides the biochemical identification, 16S rRNA sequence analyses (Microbial ID Inc., Newark, DE, USA) was carried out.

### 2.2. Bile Salts Tolerance

The method of Gilliland et al. [29], with some modifications, was used to determine bile salt tolerance. Tryptic Soy Broth (TSB) (Becton Dickinson and Co., Sparks, MD, USA) containing 0%, 0.037%, 0.075%, 0.15%, and 0.3% of bile salts number 3 (Catalog number 213010, Becton Dickinson and Co., Sparks, MD, USA) was inoculated with 10^7^ cfu/mL of each potential probiotic strain, after being centrifuged at 3000 g for 15 minutes and washed three times from their 24 h growth cultures. Samples were incubated for 24 h at 37°C with shaking at 100 rev./min. Growth in control (no bile salts) and test cultures was evaluated at 2, 4, and 24 hours by streaking samples on Tryptic Soy Agar (TSA) for presence or absence of growth. 

### 2.3. Antibiotic Resistance

Selected colonies of NP122, AM0904, B2, RW41, AM0902, AM1109A, and AM1109B on TSA plates were inoculated and cultured for 24 h in TSB at 37°C. Strains were then sent to a Veterinary Diagnostic Laboratory (University of Arkansas, Division of Agriculture, Fayetteville, AR, USA) for antibiotic sensitivity analysis using the Kirby-Bauer methodology. The diameter of the inhibition zones and the interpretative zone sizes were reported. Twelve antibiotics were tested and their concentrations were reported as shown in Table 5. The results were expressed in terms of resistant, intermediate (somewhat susceptible with zone of inhibition measuring in between of a susceptible and resistant colony), and susceptible.

### 2.4. Resistance in Conditions of the Intestinal Tract Evaluation: pH, Temperature, and Sodium Chloride

A basal TSB medium was used in these series of *in vitro* studies. A 24 h culture of each isolate was used as the inoculum whereby the cells were spun down and resuspended in 0.9% sterile saline. Then, 100 *μ*L of the suspension was inoculated into 10 mL of TSB of each test tube. Two incubation time points, that is, two and four hours were evaluated for each of the variables (pH, temperature, and NaCl). The rationale for these two points was mainly based on the transit time of food matter in the gastrointestinal tract of poultry. The temperatures tested were 15 and 45°C. The concentrations of NaCl tested were 3.5 and 6.5% (w/v). The isolates were tested for growth at pH 2 and 3. The tubes were incubated with reciprocal shaking, at the specific test temperatures or at 37°C for the tests on pH and concentrations of NaCl. At the time points evaluated, each sample was streaked on TSA for presence or absence of growth, to confirm livability of the strains. The turbidity of each tube was also noted as an indication of growth or no-growth. Each treatment was tested with triplicate tubes.

### 2.5. *Salmonella* Typhimurium *In Vivo* Growth Inhibition

A poultry isolate of *Salmonella enterica *subspecies* enterica* serovar Typhimurium (ST), which had previously been selected for resistance to nalidixic acid (NA – catalog number N-4382, Sigma, St. Louis, MO, USA), was used in all experiments. The amplification and enumeration protocol for this isolate has been described previously [30]. Trials were conducted with day-of-hatch broiler chicks obtained from a local hatchery, with the exception of one trial that was conducted with six- to seven-week-old broiler chickens. In all trials, broiler chickens were randomly (*n* = 20) assigned to untreated control diet or dietary treatment of each *Bacillus* spp. isolate at 10^5^ cfu/g of feed for seven days. Broiler chicks were housed in brooder batteries or floor pens with food and water *ad libitum. *At day four, all birds were challenged with 2 × 10^5^ cfu ST/bird. At seven days, birds were humanely killed by CO_2_ inhalation and crop, ceca, and cecal tonsils were aseptically harvested. *Salmonella *recovery procedures have been previously described by our laboratory and were followed with some modifications [30]. All animal handling procedures were in compliance with the Institutional Animal Care and Use Committee (IACUC) at the University of Arkansas.

### 2.6. Statistical Analysis

Crop and ceca colony-forming units (cfu) data were converted to log_10_ cfu numbers and then compared using the GLM procedure of SAS [31] with significance reported at *P* < 0.05. The incidence of ST recovery within experiments was compared using the chi-square test of independence [32] to determine significant (*P* < 0.05) differences between control and treated group. All values were converted to percent ST reduction comparing treated birds to nontreated birds (control) to be simplified in a single table.

## 3. Results and Discussion

### 3.1. Biochemical Tests and Identification of Selected *Bacillus* Strains

As described by Logan and Berkeley [33], the API 50 CHB system is a rapid and accurate test of *Bacillus* isolate identification, which allows bacterial isolates to be classified according to their ability to ferment 49 different carbohydrates, which are listed in Table 1. Selected *Bacillus* isolates were tested to evaluate their biochemical profile, and the results are presented in Table 1. The carbohydrate fermentation pattern was used to identify each isolate's species. Four isolates were characterized as *Bacillus subtilis/amyloliquefaciens*, and the three remaining isolates were characterized as *Bacillus licheniformis*, *Bacillus pumilus*, and *Bacillus megaterium* (Table 2). Sequence analysis of 16S rRNA is the predominant molecular technology presently available for microbial identification [34]. The 16S rRNA analysis matched the biochemical identification results (Table 2).

### 3.2. Bile Salts Tolerance

In general, tolerance to bile salts has been considered a prerequisite for colonization and metabolic activity of bacteria in the host's intestine [35]. The average concentration of bile salts in the small intestine is around 0.2% to 0.3% and may go up to 2% (w/v), depending upon the individual and the type and amount of food ingested [36, 37]. Nevertheless, bile levels in the intestine are not constant and are relatively low until ingestion of a fatty meal [38]. The main purpose of bile secretion is to emulsify and dissolve ingested fats [36]. However, bile salts also have bactericidal effects; they can disrupt the lipid membrane, get into the bacterial cell, denature proteins, chelate ions, and damage DNA [36, 39]. According to Begley et al. [38], many studies have shown that bile tolerance is a strain-specific characteristic and the tolerance of various bacterial species cannot be generalized. Also, Gram-positive bacteria seem to be more sensitive to the harmful effects of bile than Gram-negative bacteria [38].

Evaluating bile salts tolerance of the vegetative cells of our selected strains, we found that all strains were able to grow when cultured at 0.037% bile salts concentration at 2 h, 4 h, and 24 h of incubation. Six of the vegetative forms of the *Bacillus* strains tested for bile resistance were not able to survive at the concentrations of 0.075%, 0.15%, and 0.3% of bile salts during the time points evaluated. The isolate B2 was the only one able to survive at 0.075%, 0.15%, and 0.3% at 2 h of incubation (Table 3). These results are in agreement with Barbosa et al. [21] findings, where vegetative cells of *Bacillus* isolates were very susceptible to bile salts at 0.2%.

Information about the bile tolerance of Gram-positive bacteria is limited. It is important to know that bacterial tolerance to bile in broth assays, as with many physiological stresses, may not reproduce *in vivo*. Because bile salts form micelles with phospholipids, they may not be free to interact with bacterial cells, and the *in vivo* antibacterial activity of bile may be lower than that observed in *in vitro* assays [38]. Exposure to different pH, temperatures, and growth environments may increase bacterial susceptibility to bile or make them more resistant. For example, an exposure of bacteria to low levels of bile salts may increase their tolerance to higher levels [38]. Also, the presence of food in the intestinal tract can affect survival because bacteria may not be exposed to bile due to the formation of microenvironments by the food particles or food constituents, which may bind to bile components, preventing damage to the bacteria [38]. Bile resistance of some isolates is related to the enzyme activity of bile salt hydrolase (BSH) that helps to hydrolyze conjugated bile, reducing its toxic effect [40]. BSH activity has most often been found in microorganisms isolated from animals' intestines or feces [41].

The *Bacillus* spore, which consists of multiple protective layers, has been described to be very resistant to different physical and chemical conditions [21], and they have been shown to survive at high concentration (usually more than 1%) of bile salts [21, 36]. The hypothesis is that *Bacillus* spp. spores, after ingestion, would germinate in distal parts of the small intestine, where the concentration of bile salts would be lower [27, 36]. More physiological analyses are necessary to establish the importance of bile tolerance of bacteria in the intestine [38]. 

### 3.3. Resistance in Conditions of the Intestinal Tract Evaluation: pH, Temperature, and Sodium Chloride

Probiotic bacteria need to survive the passage through the stomach, where the pH can be as low as 1.5 to 2.0 [42], and stay alive for 4 h or more [43], before they move to the intestinal tract. For this reason, the vegetative cells of the isolates were evaluated for conditions similar to those found in the stomach. The isolates AM1109A and B2 were able to survive at pH 2 and pH 3 for 2 h and 4 h of exposure. On the other hand, AM0904 and AM1109B did not survive the harsh pH conditions (Table 4). The remaining isolates (NP122, AM 0902, and RW41) were able to survive at pH 2 and pH 3 at only 2 h of exposure. 

According to Ibourahema et al. [44], the bacterial capability to grow at high temperature is a good characteristic as it could be interpreted as indicating an increased rate of growth. Moreover, a high fermentation temperature reduces contamination by other microorganisms [44]. All strains grew at 15°C to 44°C at both times of incubation 2 h and 4 h (Table 4). All strains (vegetative cells) were also able to tolerate high osmotic concentrations of NaCl (Table 4). This examination gave an indication of the osmotolerance level of the *Bacillus* spp. strains. Bacterial cells cultured in a high salt concentration could have a loss of turgor pressure, which would then affect their physiology, enzyme activity, water activity, and metabolism [44]. 

### 3.4. Antibiotic Resistance

The antibiotic resistance and susceptibility of the seven *Bacillus* isolates to twelve antibiotics were analyzed. All isolates were resistant to bacitracin and sensitive to gentamycin, neomycin, ormetoprim, triple sulfa, and spectinomycin. The isolate AM0902 was also resistant to clindamycin, ceftiofur, novobiocin, penicillin, and tetracycline. The isolate RW 41 also showed resistance to erythromycin, clindamycin, ceftiofur, and novobiocin, to which B2 was resistant as well. An intermediate susceptibility was observed with AM0902 on erythromycin and with AM0904 on tetracycline (Table 5). 

According to Bakari et al. [37], probiotic bacteria that show resistance to a specific antibiotic can be given at the time of antibiotic treatment. Because antibiotic resistant genes are generally carried on conjugative plasmids, they can be transferred to other bacteria [45] and could possibly result in antibiotic-resistant enteropathogenic bacteria. Therefore, it is also important to determine whether antibiotic-resistant genes are present on chromosomes or on plasmids [37].

### 3.5. *Salmonella* Typhimurium *In Vivo* Growth Inhibition

According to Dodgson and Romanov [46], chickens have been a valuable model for human diseases and genetic analysis. Several spore-forming *Bacillus* spp. have been shown to reduce food-borne pathogens using commercial products available in Europe [2]. 

Our results showed that some *Bacillus* isolates, more specifically the isolates NP122 and the combination of the isolates AM1109A with AM1109B, were able to significantly reduce ST levels in the crop and in the ceca of broiler chickens (Table 6). The ability of *Bacillus subtilis* probiotic isolates in reducing *Salmonella *in chickens has been described previously by La Ragione and Woodward [47] and Vilá et al. [48].

Competitive exclusion of pathogens is a common hypothesis to explain the action of probiotics [49, 50]. This process has been well demonstrated in *Lactobacillus* spp., and some evidence exists that *Bacillus* spp. may have the same mode of action [21]. Competitive exclusion includes the competition for receptor sites and nutrients and the production of antimicrobial substances such as bacteriocins, hydrogen peroxide, and volatile fatty acids [49, 51]. Another potential mechanism of action of probiotics, that has received a lot of attention, is the modulation of the host's immune system [51]. According to Ng et al. and Rupa and Mine [51, 52], the probiotics alter immune functions in humans and animals by interacting with various receptors. An example is in the treatment of inflammatory bowel disease with probiotics in humans. Following probiotic treatment there are improvement of the epithelial and mucosal barrier function, modulation of the intestinal microbiota, and a direct effect on immune cells of both innate and adaptive immune systems. Despite the beneficial effects of the probiotics observed, *in vivo* mechanisms of action have not been clearly elucidated and will be a significant area for future research [53]. Several studies have shown that either live vegetative cells or spores of some *Bacillus* isolates can prevent colon carcinogenesis [18] or release antimicrobial substances against bacteria, such as *Staphylococcus aureus*, *Enterococcus faecium*, and *Clostridium difficile *[19]. These results supported the evidence of colonization and antimicrobial activity of *Bacillus* spp. as probiotic bacteria. Therefore, products containing *Bacillus* spores are used commercially as probiotics [1, 21, 22, 54–59].

## 4. Conclusion

Bacterial spore formers, especially of the genus *Bacillus*, are present in current probiotic products that have been shown to prevent gastrointestinal diseases in animals and humans. These probiotic-based spores have been shown to have many applications such as treating immunosuppressive and antibiotic-associated diarrhea. The results obtained in this study showed the tolerance of probiotic *Bacillus* spp. strains in different physiological conditions as well as the inhibition of *Salmonella* Typhimurium. Moreover, the methods used to screen isolates may be important in the evaluation of *Bacillus *spp. for use as probiotics for humans and animals.

## Figures and Tables

**Table 1:** Metabolization of different carbohydrates sources by selected isolates of *Bacillus* spp.∗

	NP122	AM0904	B2	RW41	AM0902	AM1109A	AM1109B
Amidon (starch)	+	+	+	+	−	+	+
Amygdalin	+	+	−	+	+	+	+
Arbutin	+	+	−	−	+	+	+
D-Adonitol	−	−	−	−	−	−	−
D-Arabinose	−	−	−	−	−	−	−
D-Arabitol	−	−	−	−	−	−	−
D-Cellobiose	+	+	+	+	+	+	+
D-Fructose	+	+	+	+	+	+	+
D-Fucose	−	−	−	−	−	−	−
D-Galactose	−	−	−	+	+	−	+
D-Glucose	+	+	+	+	+	+	+
D-Lactose	−	+	+	+	−	+	+
D-Lyxose	−	−	−	−	−	−	−
D-Maltose	+	+	+	+	+	+	+
D-Manitol	+	+	+	+	+	+	+
D-Mannose	+	+	+	+	+	+	+
D-Melezitose	−	−	−	−	−	−	−
D-Melibiose	−	+	+	+	−	+	+
D-Raffinose	−	+	+	+	−	+	+
D-Ribose	+	+	+	+	+	+	+
D-Saccharose	+	+	+	+	+	+	+
D-Sorbitol	+	+	+	+	−	+	+
D-Tagatose	−	−	−	+	+	−	−
D-Trehalose	+	+	+	−	+	+	+
D-Turanose	−	−	−	+	+	−	+
Dulcitol	−	−	−	−	−	−	−
D-Xylose	−	+	+	+	+	+	+
Erythritol	−	−	−	−	−	−	−
Esculin (ferric citrate)	+	+	+	+	+	+	+
Gentiobiose	+	−	−	+	−	−	−
Glycerol	+	+	+	+	+	+	+
Glycogen	+	+	+	+	−	+	+
Inositol	+	+	+	+	−	+	+
Inulin	−	−	−	+	−	−	ND
L-Arabinose	+	+	+	+	+	+	+
L-Arabitol	−	−	−	−	−	−	−
L-Fucose	−	−	−	−	−	−	−
L-Rhamnose	−	−	−	+	−	−	−
L-Sorbose	−	−	−	+	−	−	−
L-Xylose	−	−	−	−	−	−	−
Methyl-*α*D-glucopyranoside	+	+	+	+	+	+	ND
Methyl-*α*D-mannopyranoside	−	−	−	−	+	−	−
Methyl-*β*D-xylopyranoside	−	−	−	−	−	−	−
N-Acetylglucosamine	−	−	−	−	+	−	−
Potassium 2-Ketogluconate	−	−	−	−	−	−	−
Potassium 5-Ketogluconate	−	−	−	−	−	−	−
Potassium gluconate	−	−	−	−	−	−	−
Salicin	+	+	+	+	+	+	+
Xylitol	−	−	−	−	−	−	+

^*^BioMerieux API 50 CHB test kit (catalog no. 50430, bioMerieux, Marcy l'Etoile, France).

Symbols: +: growth; −: no growth. ND: not determined.

**Table 2:** Identification (ID) of *Bacillus *spp. isolates by bioMerieux API 50 CHB∗ and 16S rRNA sequence analyses∗∗.

*Bacillus* isolates	API 50 CHB identification (% ID)	16 S identification (% ID)
NP122	*Bacillus subtilis/amyloliquefaciens* (98.2%)	*Bacillus amyloliquefaciens* (96%)
AM0904	*Bacillus subtilis/amyloliquefaciens* (96.6%)	*Bacillus amyloliquefaciens* (99.57%)
B2	*Bacillus subtilis/amyloliquefaciens* (99.7%)	*Bacillus amyloliquefaciens* (99.52%)
RW41	*Bacillus licheniformis* (99.9%)	*Bacillus licheniformis* (98.66%)
AM0902	*Bacillus pumilus* (99.9%)	*Bacillus pumilus* (100%)
AM1109A	*Bacillus subtilis/amyloliquefaciens* (96.6%)	ND
AM1109B	*Bacillus megaterium* (75.3%)	ND

^*^BioMerieux API 50 CHB test kit (catalog no. 50430, bioMerieux, Marcy l'Etoile, France).

^**^16S rRNA sequence analyses (Microbial ID Inc., Newark, DE, USA).

ND: not determined.

**Table 3:** *Bacillus* spp. isolates bile salt tolerance after 2, 4, and 24 hours of incubation.

*Bacillus* isolates	0%			0.037%			0.075%			0.15%			0.3%		
	2 h	4 h	24 h	2 h	4 h	24 h	2 h	4 h	24 h	2 h	4 h	24 h	2 h	4 h	24 h
NP122	+	+	+	+	+	+	+	−	−	−	−	−	−	−	−
AM0904	+	+	+	+	+	+	−	−	−	−	−	−	−	−	−
AM0902	+	+	+	+	+	+	−	−	−	−	−	−	−	−	−
AM1109A	+	+	+	+	+	+	−	−	−	−	−	−	−	−	−
AM109B	+	+	+	+	+	+	−	−	−	−	−	−	−	−	−
RW41	+	+	+	+	+	+	−	−	−	−	−	−	−	−	−
B2	+	+	+	+	+	+	+	−	−	+	−	−	+	−	−

Symbols: +: tolerant; −: nontolerant.

**Table 4:** Effect of pH, temperature, and sodium chloride (NaCl) on the *Bacillus* spp. isolates.

*Bacillus* isolates	pH2		pH3		15°C		45°C		3.5% NaCl		6.5% NaCl	
	2 h	4 h	2 h	4 h	2 h	4 h	2 h	4 h	2 h	4 h	2 h	4 h
NP122	+	−	+	−	+	+	+	+	+	+	+	+
AM0904	−	−	−	−	+	+	+	+	+	+	+	+
AM0902	+	+	+	−	+	+	+	+	+	+	+	+
AM1109A	+	+	+	+	+	+	+	+	+	+	+	+
AM1109B	−	−	−	−	+	+	+	+	+	+	+	+
RW41	+	−	+	−	+	+	+	+	+	+	+	+
B2	+	+	+	+	+	+	+	+	+	+	+	+

Symbols: +: tolerant; −: nontolerant.

**Table 5:** Antibiotic sensitivity test∗ for *Bacillus* spp. isolates.

Antibiotics	Concentration	AM0902	AM1109A	AM1109B	AM0904	NP122	RW41	B2
Bacitracin	10 IUI/IE/U	R	R	R	R	R	R	R
Erythromycin	15 *μ*g	I	S	S	S	S	R	S
Gentamycin	10 *μ*g	S	S	S	S	S	S	S
Clindamycin	2 *μ*g	R	S	S	S	S	R	S
Ceftiofur	30 *μ*g	R	S	S	S	S	R	S
Neomycin	30 *μ*g	S	S	S	S	S	S	S
Novobiocin	5 *μ*g	R	S	S	S	S	R	R
Penicillin	10 IUI/ IE/U	R	S	S	S	S	S	S
Ormetoprim	1.25 *μ*g	S	S	S	S	S	S	S
Tetracycline	30 *μ*g	R	S	S	I	S	S	S
Triple sulfa	1.0 mg	S	S	S	S	S	S	S
Spectinomycin	100 *μ*g	S	S	S	S	S	S	S

^*^Veterinary Diagnostic Laboratory (University of Arkansas, Division of Agriculture, Fayetteville, AR, USA).

R: resistant; I: intermediate; S: susceptible.

**Table 6:** Effect of *Bacillus* spp. isolates in reducing *Salmonella* Typhimurium from crop and ceca of broiler chickens in an avian model.

*Bacillus* isolates	Crop % reduction	Crop log 10 reduction	Cecal tonsils % reduction	Ceca log 10 reduction
NP 122	15.8	ND	50	2.5∗
AM 0904	0	ND	ND	0
RW 41	0	ND	ND	0
B2	0	ND	ND	0
AM 1109 A and B	8.4	1.62∗	ND	ND
AM 1109 A and B (6-7-week-old broilers)	10	0.63	15.8	1.15∗

^*^Significantly different at *P* < 0.05.

ND: not determined.
